# The role of lifestyle factors in the association between early-life stress and adolescent psycho-physical health: Moderation analysis in two European birth cohorts

**DOI:** 10.1016/j.ypmed.2024.107926

**Published:** 2024-03-04

**Authors:** Serena Defina, Tom Woofenden, Vilte Baltramonaityte, Henning Tiemeier, Graeme Fairchild, Janine F. Felix, Charlotte A.M. Cecil, Esther Walton

**Affiliations:** a Department of Child and Adolescent Psychiatry, Erasmus MC, University Medical Center Rotterdam, Rotterdam, the Netherlands; b Generation R Study Group, Erasmus MC, University Medical Center Rotterdam, Rotterdam, the Netherlands; c Department of Psychology, University of Bath, Bath, United Kingdom; d Department of Social and Behavioral Sciences, T.H. Chan School of Public Health, Harvard University, Boston, MA, USA; e Department of Epidemiology, Erasmus MC, University Medical Center Rotterdam, Rotterdam, the Netherlands; f Department of Paediatrics, Erasmus MC, University Medical Center Rotterdam, Rotterdam, the Netherlands; g Molecular Epidemiology, Department of Biomedical Data Sciences, Leiden University Medical Center, Leiden, the Netherlands

**Keywords:** Adverse childhood experiences, Comorbidity, Physical activity, Sleep, Diet, Mediterranean, Depression, Adiposity, Moderation analysis

## Abstract

**Objective:**

Early-life stress (ELS) is an established risk factor for a host of adult mental and physical health problems, including both depression and obesity. Recent studies additionally showed that ELS was associated with an increased risk of comorbidity between mental and physical health problems, already in adolescence. Healthy lifestyle factors, including physical activity, sleep and diet have also been robustly linked to both emotional and physical wellbeing. However, it is yet unclear whether these lifestyle factors may moderate the association between ELS and psycho-physical comorbidity.

**Methods:**

We investigated whether *(a)* participation in physical activity, *(b)* sleep duration, and *(c)* adherence to a Mediterranean diet, moderated the relationship between cumulative ELS exposure over the first 10 years of life and psycho-physical comorbidity at the age of 13.5 years. Analyses were conducted in 2022–2023, using data from two large adolescent samples based in the UK (ALSPAC; *n* = 8428) and The Netherlands (Generation R; *n* = 4268).

**Results:**

Exposure to ELS was significantly associated with a higher risk of developing comorbidity, however this association was not modified by any of the three lifestyle factors investigated. Only physical activity was significantly associated with a reduced risk of comorbidity in one cohort (OR_*ALSPAC*_ [95%CI] = 0.73 [0.59;0.89]).

**Conclusions:**

In conclusion, while we found some evidence that more frequent physical activity may be associated with a reduction in psycho-physical comorbidity, we did not find evidence in support of the hypothesised moderation effects. However, more research is warranted to examine how these associations may evolve over time.

## 1 Introduction

Over the past few decades, the prevalence of comorbid mental and physical diseases has risen dramatically, posing a major challenge for health services across the world (Launders et al., 2022; Ronaldson et al., 2021). In particular, multiple large studies have shown a substantial degree of comorbidity between common mood disorders, such as depression and anxiety, and cardio-metabolic conditions, including diabetes, obesity and cardiovascular disease (Fisher et al., 2014; Gold et al., 2020; Souama et al., 2023). Mounting evidence is further suggesting an early origin of these psycho-physical comorbidity patterns, involving shared risk factors and pathophysiological pathways that begin already in utero (Milaneschi et al., 2019). One of such risk factors is psychosocial stress – including for instance family conflict, financial difficulties or victimization, experienced in the first years of life – here collectively defined as early-life stress (ELS). For example, a recent study conducted in two independent population-based samples reported that exposure to ELS during gestation and throughout the first 10 years of life prospectively associated with increased internalizing symptoms,^2^ adiposity, as well as their co-occurrence in adolescence (Defina et al., 2023). The study focused on broad, pre-clinical measures of depression/anxiety (i.e. internalizing symptoms) and cardio-metabolic risk (i.e. adiposity) respectively. However, these associations were also shown to persist into mid- and late adulthood and manifest into clinical diagnoses (Bright et al., 2016; Milaneschi et al., 2019; Souama et al., 2023), highlighting the early developmental origins of these risk pathways.

While these findings certainly support the importance of primary prevention programmes aimed at reducing the incidence of ELS, preventing ELS may not always be possible. As such, there is a need to identify alternative modifiable factors that could mitigate the negative impact of ELS on later health, and inform the development of complementary intervention strategies.

Healthy lifestyle factors, including physical activity, sleep and diet have been robustly linked to both emotional and physical wellbeing (Briguglio et al., 2020; Firth et al., 2020). For example, regular physical activity was associated with reduced internalizing (Wheatley et al., 2020) and depressive symptoms (Oberste et al., 2020), as well as with lower body mass index (BMI) (Schwarzfischer et al., 2017) in children and adolescents. Adolescents reporting longer sleep durations have also been shown to be at lower odds of developing depression and obesity (Owens et al., 2014). Moreover, diet quality, particularly adherence to a Mediterranean diet, was associated with reduced internalizing symptoms (Orlando et al., 2022) and risk of depression (Shafiei et al., 2023), as well as with lower adiposity (Tognon et al., 2014) in childhood.

However, it remains unclear whether any of these lifestyle factors could effectively attenuate the association between ELS exposure and the risk of comorbidity between mental and physical health problems, in early adolescence.

To address this gap, we replicated and extended Defina et al. (2023) approach, by investigating the interaction between cumulative ELS exposure and *(a)* participation in physical activity, *(b)* sleep duration, and *(c)* adherence to a Mediterranean diet, on adolescent psychophysical comorbidity, defined as the co-occurrence of high internalizing symptoms and high adiposity.

## 2 Methods

This study follows STROBE guidelines (von Elm et al., 2008).

## 3 Participants

Our sample was drawn from two ongoing population-based longitudinal birth cohorts: the Avon Longitudinal Study of Parents and Children (ALSPAC) involving a total of total of 14,833 pregnant women whose children were born in Avon (UK) between 1991 and 1992 (Boyd et al., 2012; Fraser et al., 2013); and the Generation R (GenR) Study, involving 9778 pregnant women, with children born in Rotterdam (the Netherlands) between 2002 and 2006 (Kooijman et al., 2016). Please note that the ALSPAC website contains details of all the data that is available through a fully searchable data dictionary and variable search tool (http://www.bristol.ac.uk/alspac/researchers/our-data/). More detailed information about ALSPAC study numbers can be found in Supplementary Text S1.

Response rates at the 13 years follow-up were 61% in ALSPAC and 64% in GenR. Participant selection criteria have been previously described (Defina et al., 2023). In summary, children with >50% missing ELS items in either the pre- or postnatal period were excluded. All twins were further excluded and, in the case of non-twin siblings, only one was retained in the sample. The final sample included 8428 and 4268 children in ALSPAC and GenR respectively.

Ethical approval for the study was obtained from the ALSPAC Ethics and Law Committee, the University of Bath Psychology Research Ethics Committee (reference number: 20–195), and from the Medical Ethical Committee of Erasmus MC, University Medical Center Rotterdam (MEC-198.782.2001.31).

## 4 Measures

### 4.1 Early Life Stress (ELS) exposure

A detailed description of the ELS exposure score can be found elsewhere (Defina et al., 2023); ELS score repository). Briefly, a prenatal (i. e., maternal exposure during pregnancy) and postnatal (i.e. from birth to 10 years) cumulative ELS score was constructed in each cohort by combining ~100 stress-related items (e.g., death of a relative; financial difficulties; parental psychopathology; maltreatment or bullying). Prenatal and postnatal stress scores were then summed to obtain a total ELS exposure score, spanning from pregnancy to age 10 years.

### 4.2 Physical and mental health outcomes

Following the same approach as Defina et al., we defined psychophysical comorbidity as the co-occurrence of high internalizing symptoms and high adiposity.

Internalizing symptoms were assessed by parental reports when children were on average 13.5 years old (range: 12.5–16.8 years) using the Strengths and Difficulties Questionnaire (SDQ) (Goodman, 1997) in ALSPAC and the Child Behaviour Checklist (CBCL 6–18) (Achenbach, 1999) in GenR. Both instruments are well-validated reports of emotional and behavioral functioning referring to the past 6 months, and have been shown to be comparable (*r* = 0.74) (Goodman and Scott, 1999).

Adiposity was measured using total body fat mass percentage estimated via dual-energy X-ray absorptiometry (DXA) scanner (ALSPAC: Lunar Prodigy DXA scanner, GE Healthcare; GenR: iDXA scanner, GE Healthcare, Madison, WI) at the average age of 13.5 years (range: 12.5–16.6 years).

Finally, to obtain psycho-physical comorbidity, internalizing symptoms and fat mass percentage were first dichotomized into high versus low-moderate, based on a cohort-specific 80th percentile cut-off value. The dichotomized values were then used to assign children to 4 groups: “*Healthy*” (both outcomes <80th percentile); “*High internalizing*” (internalizing >80th & fat mass percentage ≤ 80th); “*High adiposity*” (internalizing ≤80th & fat mass percentage > 80th); and “*Comorbid*” (both outcomes >80th percentile). Note that the 80th percentile cut-off was based on previous validation studies (Achenbach and Rescorla, 2001; Bourdon et al., 2005; Flegal et al., 2010; Weber et al., 2014) and the resulting size of the comorbidity group was larger than expected by chance (permutation test *p* < 0.01; see Supplement 2 in Defina et al., 2023).

### 4.3 Lifestyle factors

#### 4.3.1 Physical activity

Frequency of physical activity was measured via questionnaires when children were on average 10.7 years old (range: 10.6–14.7) in ALSPAC and 9.7 years (range: 8.9–12.4) in GenR. ALSPAC mothers were asked about the “Average number of times their child participated in vigorous physical activity in past month” (“none”, “less than once a week”, “1-3 times a week”, “4-6 times a week” and “daily”), while GenR children reported “How often did they play sports at a sports club or team” (“once a week”, “2 times a week”, “3 times a week”, “4 times a week”, or “5 or more times a week”).

#### 4.3.2 Sleep

In the ALSPAC sample, sleep duration (hours) was calculated using maternal reports of the “Time their child usually goes to sleep and wakes up on normal school days”, collected at the mean age of 11.7 years (range: 11.4–13.8).

**Fig. 1:**
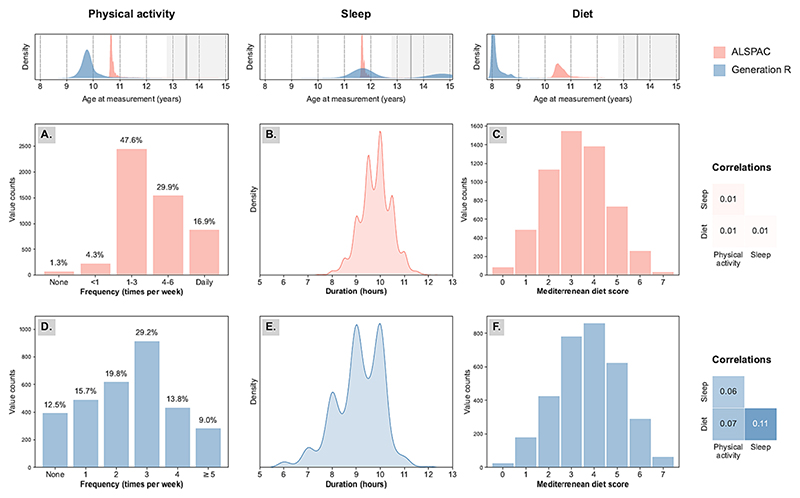
Lifestyle factors characteristics in ALSPAC and Generation R children (UK, 2002–2005; The Netherlands, 2010–2016). Characteristics of physical activity, sleep and diet measurements in the ALSPAC (A-C; in pink) and GenR cohorts (D-F; in blue). In the top panel, the distributions of child age at moderator measurement are depicted for both cohorts. A grey line and shadow also indicate the average and range of child age at outcome measurement. The central and bottom panel depict the distribution and correlation between the three lifestyle factors in ALSPAC and GenR respectively. (For interpretation of the references to colour in this figure legend, the reader is referred to the web version of this article.)

In GenR, a selected subsample of children (*N* = 1483; (Koopman-Verhoeff et al., 2019) completed a sleep diary including questions about the time they went to bed and woke up, on nine consecutive days (i.e., 5 weekdays and 4 weekend days). These self-reports were used to calculate average sleep duration across the nine days. Mean age of measurement was 12.7 years (range: 10.4–15.6), however, due to logistical reasons, data collection was split into two waves, resulting in a 3-years age difference between the first and second group (i.e., 11 and 14 years; see Fig. 1B). Sleep duration values measured after either outcome of interest were set to missing (*N* = 519).

#### 4.3.3 Diet

Nutritional intake was assessed at 10.6 years (range: 9.8–12.2) in ALSPAC and at 8.2 years (range: 7.5–10.8) in GenR, using a 3-day child-reported food diary (Cribb et al., 2011), and the 4-week parent-reported food frequency questionnaire (FFQ) (Dutman et al., 2011), respectively.

ALSPAC participants were asked to record all foods and drinks consumed over three individual days (preferably one weekend and two weekdays, not necessarily consecutive), using household measures. The structured diary was designed for the child to complete with the help of their parents. They would then bring the diary to the clinic visit where, if possible, any uncertainties were clarified by a member of the nutrition team. Food records were transformed into food codes and associated weights using the DIDO software (Price et al., 1995).

The FFQ consists of 71 food items relevant for the energy intake of Dutch children (Netherlands Nutrition Centre, E, 1998). Information on frequencies, types, and portion sizes was converted into grams of individual food items per day based on standard Dutch portion sizes (van der Velde et al., 2019).

A Mediterranean diet adherence score (ranging from 0 to 7) (Trichopoulou et al., 2003) was constructed by assigning 1 point for the elevated (i.e., ≥ median) consumption of five beneficial food groups (i. e., vegetables, legumes, fruits and nuts, cereal, and fish), and 1 point for the restricted (i.e., < median) consumption of two detrimental food groups (i.e., meat and dairy products). Details of specific items included in the food groups in each cohort can be found in Supplementary Text S2.

## 5 Statistical analysis

Analyses were run separately in the two cohorts, using R version 4.1.0 (R Core Team, 2021, 2024) (scripts publicly available at https://github.com/SereDef/lifestyle-moderators-project).

Missing values in all variables of interest (i.e., exposures, outcomes, moderators and covariates) were imputed by conditional multiple imputation (Buuren and Groothuis-Oudshoorn, 2011) using 60 iterations and 30 imputed datasets (for a complete assessment of missing values and detailed imputation strategy see Supplementary Text S3 and Supplementary Table S1). Model parameters were fit in each imputed dataset and then pooled according to Rubin’s Rules. To account for multiple comparisons, false discovery rate correction was applied.

### 5.1 Main analyses

To address our primary hypotheses, three multinomial logistic regressions (i.e., one for each lifestyle factor) were performed, with psycho-physical risk group as a dependent variable. The reference level was set to “healthy”. These models included: *a)* the main effect of total ELS, *b)* that of each lifestyle factor (separately; i.e., physical activity, sleep and diet) and *c)* their interaction, as well as the full set of covariates. In order to generate meaningful and comparable estimates for the main effects of interest, both ELS scores and lifestyle factor variables were standardized using a z transformation before entering the models. The covariate set included child sex, age at outcome measure, ethnicity (dichotomized into “White” and “non-White”), maternal smoking and alcohol consumption during pregnancy and maternal pre-pregnancy BMI. To diagnose multicollinearity between independent variables we computed generalized variance inflation factors (VIF) for each predictor (James et al., 2013).

### 5.2 Follow-up analyses

Several follow-up analyses were conducted, whereby the three main models (i.e., for physical activity, sleep and diet) were modified to:

a) Feature *(i)* internalizing symptoms and *(ii)* adiposity (rather than their comorbidity) as dependent variables (i.e., in two separate linear regression models);
b) Examine pre–/postnatal ELS (rather than the total ELS score) as main stress exposure;
c) Assess the effect of adhering to international guidelines regarding weekly physical activity, sleep duration and diet, by using a dichotomized version of each moderator.

We dichotomised the physical activity variable according to the WHO recommended guideline for vigorous physical activity of “at least 3 times a week” for children aged 5–17 (Bull et al., 2020). Applied to available response options in both cohorts, participation in physical activity was deemed “infrequent” (0–3 times a week) or “frequent” (4+ times a week).

The sleep variable was dichotomized based on the recommendations of the ‘American Academy of Sleep Medicine’ for children aged 6–12 years (Paruthi et al., 2016). Children who slept between 9 and 12 h were categorised as sleeping a “recommended” duration, in comparison to “insufficient/excessive” sleep. Finally, we used a median-split approach to dichotomise the diet variable. Children were categorised as having lower (≤ median), or higher adherence (> median) in each sample.

We additionally explored potential non-linear associations between each lifestyle factor and the main outcomes of interest by including second- and third-degree polynomial terms in the models. To assess the extent to which maternal health and lifestyle behaviour may have moderated the relationships of interest (Epstein et al., 2023; Heslehurst et al., 2019), we further investigated the interaction between ELS and maternal BMI on child comorbidity. Finally, to assess the impact of the imputation procedure on our results, we ran the analyses in the subsample with complete moderator and outcome data.

## 6 Results

### 6.1 Sample characteristics

Sample characteristics were pooled across imputed datasets and summarized in Table 1. Briefly, the ALSPAC sample included 8428 (48% male) children, whose mothers were 96% White and 30% highly educated (i.e., held a college or university degree). The GenR sample included 4268 (52% male) participants, 71% of which were encoded as “White” (i.e., European, North American, Japanese or Indonesian) and 14% had highly educated (i.e., “higher, phase 2”) mothers. The distribution of each lifestyle factor and their correlations are displayed in Fig. 1. The proportion of children categorised as comorbid was small (2.9% in ALSPAC and 5.1% in GenR), as also reflected by the weak correlations between internalizing scores and adiposity (*r* = ALSPAC: 0.10; GenR: 0.12; see Supplementary Table S2).

### 6.2 Main analyses

**Fig. 2:**
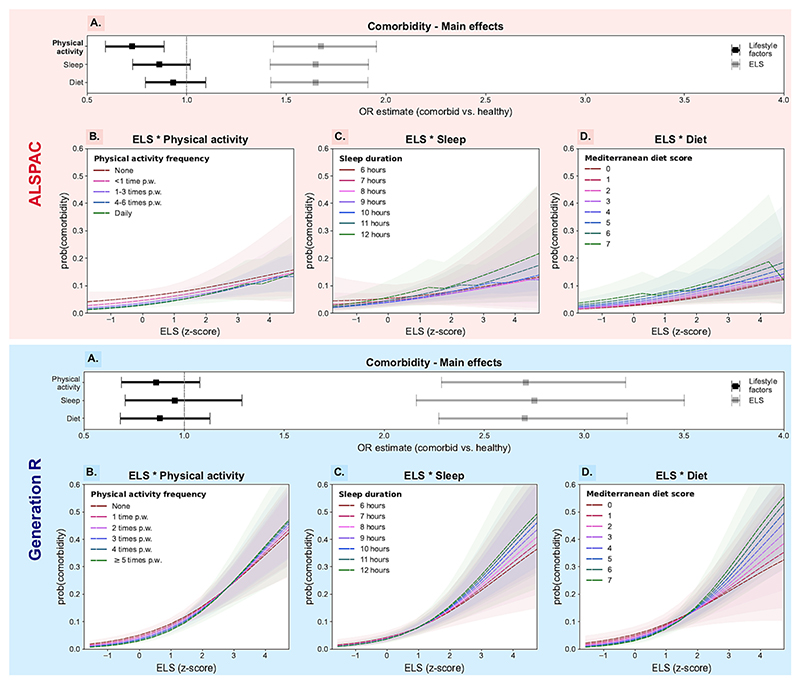
Main effects and interactions between lifestyle factors and early-life stress on comorbidity risk in ALSPAC and Generation R children (UK, 1992–2006; The Netherlands, 2002–2016). A. Odd ratios (OR) and their 95% confidence intervals (CI) are represented along the x-axis for the main effects of physical activity, sleep and diet (in black) and for that of ELS (in grey). Statistically significant terms are highlighted in bold. B–D. The predicted probability of developing comorbidity (y-axis) against ELS exposure levels (x-axis), stratified by lifestyle factor levels (red to green coloured lines). The 95% CIs around the predicted probabilities are also shown for each line (i.e., lifestyle factor level) and their overlap is an indication for the lack of interaction between ELS and physical activity, sleep or diet. (For interpretation of the references to colour in this figure legend, the reader is referred to the web version of this article.)

We did not find evidence in support of any of the hypothesised moderation effects (Supplementary Table S3). Increased ELS was significantly associated with higher risk of developing comorbidity (vs. being healthy; Fig. 2A; ORs: 1.65–1.67 in ALSPAC and 2.70–2.75 in GenR), but this association was not modified by any of the three lifestyle factors we investigated (Fig. 2B-D).

Conversely, while comorbidity risk was generally lower in children who engaged in favourable lifestyle behaviours (Fig. 2A), this effect was only significant for physical activity and only in one of the two cohorts (OR_*ALSPAC*_ [95%CI] = 0.73 [0.59;0.89]). The magnitude of multicollinearity was low in all the models (VIF ≤ 1.17; see Supplementary Table S3).

## 7 Follow-up analyses

**Fig. 3:**
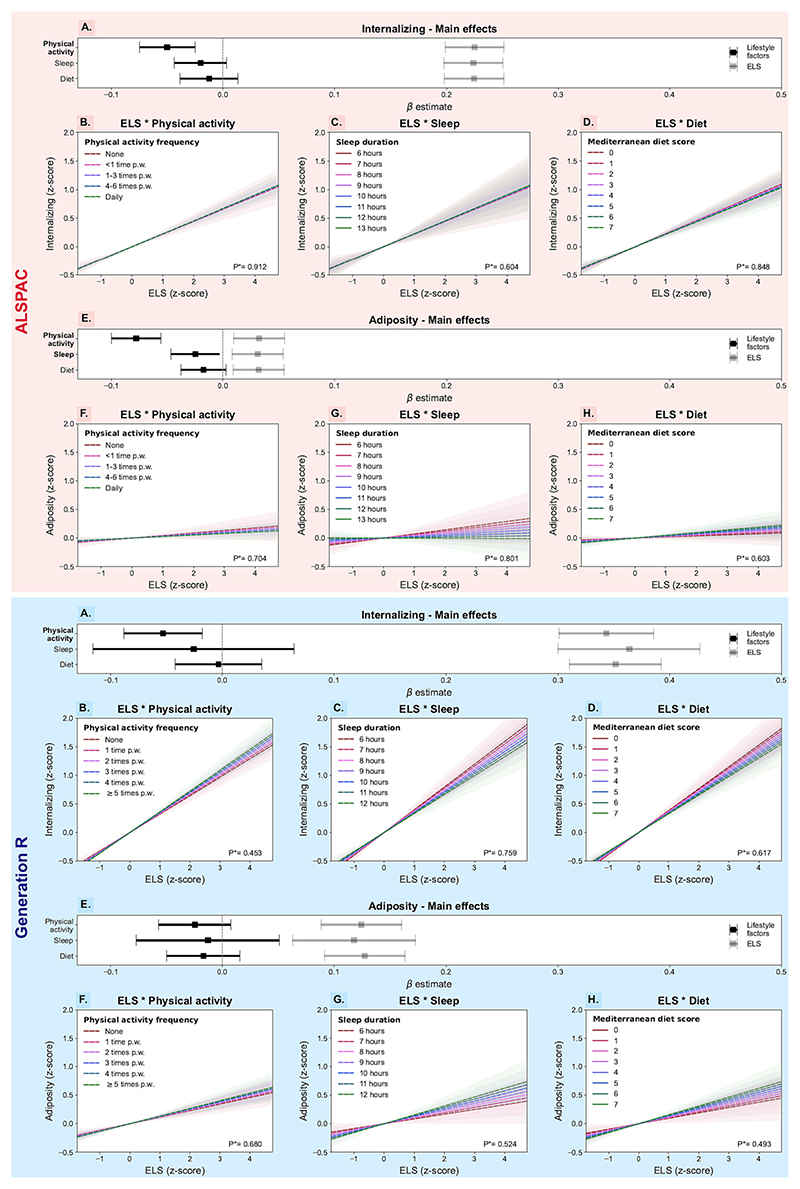
Main effects and interactions between lifestyle factors and early life stress on internalizing symptoms and adiposity in ALSPAC and Generation R children (UK, 1992–2006; The Netherlands, 2002–2016). A, E. For each outcome (A. Internalizing symptoms; E. Adiposity), the standardized effect estimates and their 95% confidence intervals (CI) are represented along the x-axis for the main effects of physical activity, sleep and diet (in black) and for that of ELS (in grey). Statistically significant terms are highlighted in bold. B–D and F–H. Linear association between ELS exposure on the x-axis and internalizing symptoms (B–D) or adiposity (F–H) on the y-axis, stratified by lifestyle factor levels (red to green coloured lines). The 95%CIs around each slope (i.e., lifestyle factor level) are also shown and their overlap is indication of the lack of interaction between ELS and physical activity, sleep or diet. *P*-value for the said interaction is also noted at the bottom right of each plot. (For interpretation of the references to colour in this figure legend, the reader is referred to the web version of this article.)

When internalizing symptoms and adiposity were examined as two separate outcomes, a similar pattern of results emerged (Supplementary Tables S4;Fig. 3). ELS was significantly associated with increased internalizing symptoms and adiposity, but neither association was modified by physical activity, sleep or diet.

Only the main effect of physical activity on internalizing symptoms was statistically significant in both cohorts (β_*ALSPAC*_ [95%CI] = −0.05 [−0.08;−0.03]; β_*GenR*_ [95%CI] = −0.05 [−0.09;-0.02]).

Engagement in two of the three favourable lifestyle behaviours was linked to lower adiposity levels, but only in one of the two cohorts (β_*ALSPAC*_ [95%CI]: physical activity = −0.08 [−0.10;-0.06]; sleep = −0.02 [−0.05;0.01]).

Findings did not change substantially when ELS exposure was restricted to the prenatal or postnatal periods only (Supplementary Table S5), nor when lifestyle factors were dichotomized into adherence and non-adherence to international guidelines (Supplementary Table S6). The non-linear relationships between each lifestyle factor and the main outcomes of interest are represented in Supplementary Fig. S1. Only for the relationship between sleep and internalizing symptoms did we find any evidence for a non-linear (inverse logarithmic) trend. We did not find a statistically significant interaction between ELS exposure and maternal BMI on child comorbidity (Supplementary Table S7). The sensitivity analysis conducted in the subsample with complete outcome and moderator data (sample size = ALSPAC: 3680–4237; GenR: 961–2369) also did not impact our main conclusions (Supplementary Table S8).

## 8 Discussion

In this study based on data from two independent population-based cohorts, we did not find evidence that either physical activity, sleep or dietary behaviour may attenuate the association between ELS exposure and adolescent psycho-physical comorbidity.

However, at average levels of stress exposure, engaging in more frequent physical activity (more so than sleeping adequately or following a Mediterranean diet) was significantly associated with a reduced risk of developing comorbidity, as well as with lower levels of both internalizing symptoms and adiposity, in one of the cohorts investigated. While the direction of effects was generally consistent across cohorts, only the association between physical activity and internalizing symptoms resulted statistically significant in both ALSPAC and GenR children. Overall, it is important to note that the association between lifestyle behaviours and psycho-physical comorbidity was considerably smaller compared to that between ELS and comorbidity.

In line with these findings, previous studies conducted in adult populations also showed how the association between retrospectively reported childhood maltreatment and higher odds of psycho-physical comorbidity, was still present after additional adjustment for concurrent lifestyle factors such as smoking, alcohol use, sleep and physical activity (Souama et al., 2023; Tomasdottir et al., 2015), although none of these previous studies formally tested for ELS-by-lifestyle interactions.

Our results also align with the previously reported protective effects of physical activity (e.g., swimming or cycling) for reducing the risk of depression (Choi et al., 2019; Choi et al., 2020; Firth et al., 2020; He et al., 2021; Schuch et al., 2018), although this was not specific to children exposed to higher levels of stress (Choi et al., 2020).

In contrast, we could not find convincing evidence that either longer sleep duration or improved diet quality would alleviate the risk for depression or cardiometabolic problems (or their combination), as suggested by some previous studies (He et al., 2021; Katikireddi et al., 2017; Lassale et al., 2019; Zhai et al., 2015). Developmental timing and/or differences in outcome measurement may have played a role in explaining this discrepancy. It is possible, for example, that engaging in healthy lifestyle behaviours later in life may be more beneficial, or that the protective effects of childhood lifestyle behaviours may manifest only later in adulthood. It is also possible that the associations reported in the adult literature may be biased through reverse causation (e.g., depression being a causal risk factor for poor diet and sleep; (Choi et al., 2020).

One further insight emerging from these analyses was that of a stronger magnitude of association between ELS and comorbidity compared to that between each of the three lifestyle factors and comorbidity. While this result could be interpreted as an indication of the more severe, long-lasting impact of ELS, as discussed elsewhere (Henchoz et al., 2019; Souama et al., 2023; Tomasdottir et al., 2015), we would like to highlight that differential measurement error could have played a role in explaining this finding. Indeed, our ELS measure was considerably more comprehensive (i.e., comprised of many more items and covering a longer time period) compared to each of the lifestyle factors, which were only assessed at a single time point and based on fewer indicators. It is possible, for example, that the hypothesised moderation effects may emerge when a more long-term engagement in physical activity is considered.

In addition to this point, a few other study limitations need to be considered when interpreting these results. First, our measurement of ELS, lifestyle behaviours and internalizing symptoms rely primarily on parent reports, which might have introduced information bias. ELS exposure was assessed over a large (~10 years) temporal window, but the measure was inevitably constrained by data availability. Additionally, cohort differences in the reporting of physical activity (i.e., “vigorous physical activity” in ALSPAC, vs. “sports” in GenR) may have contributed to heterogeneous findings for the main effect of physical activity on comorbidity. For example, “sports” can include non-vigorous activities, which are potentially less effective in reducing depressive symptoms (Oberste et al., 2020). Second, while we focused here on three lifestyle markers that are most relevant to the developmental period of interest and may constitute important targets for early prevention, other potentially relevant factors such as smoking, alcohol use or psychological coping strategies, are also likely to be of relevance (Hanlon et al., 2020; Skou et al., 2022) and should be taken into account in future studies. Further, although we adjusted our analyses for several important confounders and we addressed the challenge of selection bias thoroughly by multiple imputation and sensitivity analyses, we cannot exclude the possibility that residual sampling bias and/or other unmeasured factors (e.g., pubertal status or (epi-)genetic influences) may have influenced our results. For instance, the majority of the children included in our samples were of European descent, and these findings may not be generalizable to different populations. Furthermore, shared biological risk factors for depression and obesity (e.g. dysregulation of the hypothalamic-pituitary-adrenal axis, chronic inflammation, or microbiome dysbiosis; (Milaneschi et al., 2019) could potentially mediate the effect of ELS on psycho-physical comorbidity. Finally, our study focused on one aspect of adolescent mental health, i.e. internalizing symptoms, that is most predictive of future depression/anxiety problems. However, other measures of adolescent behaviour, e.g., externalizing problems, could represent interesting targets for future studies in the field.

In conclusion, several international guidelines and policies already acknowledge the importance of lifestyle interventions not only for the obesity and poor cardiovascular health crisis, but also with respect to their protective effects against poor mental health (Australian Department of Health, 2014; Department of, H., Social, C, 2011; National Institute for, H., Care, E, 2015; Stubbs et al., 2018; Tremblay et al., 2016). Although the current study reports a lack of influence from lifestyle factors, a non-significant interaction should not be interpreted as evidence that adopting a healthy lifestyle is ineffective in mitigating the detrimental consequences of ELS on comorbid internalizing and adiposity in childhood. Instead, our study further emphasizes the need for the investigation of early comorbidity prevention strategies that target children who have experienced ELS, and that more research is warranted to examine the potential moderating effects of a wider range of lifestyle behaviours.

## 9 Conclusions

Our study could not detect convincing evidence in support of the hypothesis that the detrimental effects of ELS on psycho-physical co-morbidity risk may be attenuated by adopting healthier lifestyle habits in childhood. We found some suggestive evidence that engaging in more frequent physical activity may reduce the risk for comorbidity, irrespective of stress exposure levels. If confirmed in future studies, this set of findings could highlight the need for more comorbidity prevention efforts focused on reducing ELS, in addition to current intervention programmes that focus on lifestyle behaviours such as physical activity.

## Supplementary Material

Supplementary Figures and Text

Supplementary tables

## Figures and Tables

**Table 1:** Descriptive statistics of ALSPAC and Generation R children (UK, 1992–2006; The Netherlands, 2002–2016).

	ALSPAC (*n* = 8428) Mean (range) / n (%)		Generation R (n = 4268) Mean (range) / n (%)	
Age at outcome		13.49 (12.79−14.96)	13.56 (12.59−16.63)	
Sex	Male	4370 (51.9)	Male	2091 (49.0)
Ethnic background	White	8103 (96.1)	White	3061 (71.7)
Early life stress		3.57 (0.43−18.50)		1.28 (0.00−6.00)
	None	128 (1.5)	None	568 (13.3)
	<1 week	377 (4.5)	Once a week	626 (14.7)
Frequency of participation in physical activity	1−3 week	3946 (46.8)	Twice a week	728 (17.1)
	4−6 week	2408 (28.6)	Three times a week	1138 (26.7)
	Daily	1566 (18.6)	Four times a week	643 (15.1)
			5 or more times a week	563 (13.2)
Sleep duration (hours)		9.81 (6.00−13.00)	8.99 (6.00−12.00)	
Mediterranean diet score		3.24 (0.00−7.00)	3.83 (0.00−7.00)	
Internalizing symptoms		1.43 (0.00−10.00)	5.75 (0.00−41.00)	
Fat mass percentage		24.36 (4.92−56.25)	25.53 (8.49−54.62)	
Comorbidity risk group	Healthy	5916 (70.2)	Healthy	2789 (65.4)
	High internalizing	795 (9.4)	High internalizing	622 (14.6)
	High adiposity	1476 (17.5)	High adiposity	637 (14.9)
	Comorbidity	241 (2.9)	Comorbidity	219 (5.1)
Maternal BMI (kg/m^2^)		22.79 (12.49−48.62)	23.46 (14.38−50.21)	
Maternal smoking	Never	4412 (52.3)	Never	3234 (75.8)
	Until pregnancy	2524 (30.0)	Until pregnancy	386 (9.0)
	During pregnancy	1492 (17.7)	During pregnancy	648 (15.2)
		0.73 (0.00−3.50)	Never	1694 (39.7)
Maternal alcohol consumption	Continuous score^1^		Until pregnancy	599 (14.0)
			Continued occasionally	1567 (36.7)
			Continued frequently	405 (9.5)
	Low	4216 (50.0)	Low	1716 (40.2)
Maternal education^2^	Medium	3001 (35.6)	Medium	1278 (29.9)
	High	1212 (14.4)	High	1274 (29.9)

Sample characteristics of each cohort, pooled across 30 imputed datasets. We report mean (range) for continuous variables and *n* (percentage) of each group for categorical variables.

1 Higher scores correspond to higher alcohol use frequencies throughout pregnancy.

2 Maternal education: Low = “none”, “certificate of secondary education”, “vocational” or “O level” in ALSPAC, “secondary, phase 2” or lower in GenR; medium = “a level” in ALSPAC, “higher, phase 1” in GenR; high = “(college or university) degree” in ALSPAC, “higher, phase 2” in GenR. Categorization based on the international standard classification of education 2011.

## Data Availability

The datasets are available upon request and subject to cohort-specific executive data access procedures. ALSPAC data access is through a system of managed open access and can be obtained from ALSPAC through the standard ALSPAC research proposal and data access policy (http://www.bristol.ac.uk/alspac/researchers/access/). Data from the Generation R Study are available upon reasonable request to the director of the Generation R Study (generationr@erasmusmc.nl), subject to local, national and European rules and regulations. ALSPAC data access is regulated through a system of managed open access. Please note that the ALSPAC study website contains details of all the data that is available through a fully searchable data dictionary and variable search tool (http://www.bristol.ac.uk/alspac/researchers/our-data/). All scripts employed in the analyses were made publicly available (https://github.com/SereDef/lifestyle-moderators-project).

## Footnotes

2 Internalizing symptoms (Achenbach, 1966) refer to a host of problems, including feelings of sadness, withdrawal, anxiety, loneliness or somatic complaints that are typical of early manifestations of depression and anxiety during childhood and adolescence.

## Abbreviations

ALSPAC: Avon longitudinal study of parents and children
BMI: Body mass index
CI: Confidence interval
CBCL: Child behaviourchecklist
DXA: Dual-energy X-ray absorptiometry
ELS: Early life stress
FFQ: Food frequency questionnaire
GenR: Generation R
OR: Odds ratio
SDQ: Strengths and difficulties questionnaire
VIF: Variance inflation factor
UK: United Kingdom.
