# Supplementary material for: The role of lifestyle factors in the association between early-life stress and adolescent psycho-physical health: Moderation analysis in two European birth cohorts
Source: Prev Med. Author manuscript; Available in PMC 2024 Jul 6. (PMC7616134; doi:10.1016/j.ypmed.2024.107926)
Supplement: Supplementary Figures and Text [file EMS196865-supplement-Supplementary_Figures_and_Text.docx]

**Supplementary Materials**

“The role of lifestyle factors in the association between early-life stress and adolescent psycho-physical health: moderation analysis in two European birth cohorts”

Table of Contents

[Supplementary text 1 | ALSPAC Study sample description 1](#_Toc151978142)

[Supplementary text 2 | Mediterranean Diet score calculation 1](#_Toc151978143)

[Supplementary text 3 | Imputation rationale and quality 2](#_Toc151978144)

[Figure S1 | Non-linear relations 5](#_Toc151978145)

[Supplementary references 6](#_Toc151978146)

# Supplementary text 1 | ALSPAC Study sample description

Pregnant women resident in Avon, UK with expected dates of delivery between 1st April 1991 and 31st December 1992 were invited to take part in the study. 20,248 pregnancies have been identified as being eligible and the initial number of pregnancies enrolled was 14,541. Of the initial pregnancies, there was a total of 14,676 fetuses, resulting in 14,062 live births and 13,988 children who were alive at 1 year of age. When the oldest children were approximately 7 years of age, an attempt was made to bolster the initial sample with eligible cases who had failed to join the study originally. As a result, when considering variables collected from the age of seven onwards (and potentially abstracted from obstetric notes) there are data available for more than the 14,541 pregnancies mentioned above: The number of new pregnancies not in the initial sample (known as Phase I enrolment) that are currently represented in the released data and reflecting enrolment status at the age of 24 is 906, resulting in an additional 913 children being enrolled (456, 262 and 195 recruited during Phases II, III and IV respectively). The phases of enrolment are described in more detail in the cohort profile paper and its update (see footnote 5 below). The total sample size for analyses using any data collected after the age of seven is therefore 15,447 pregnancies, resulting in 15,658 fetuses. Of these 14,901 children were alive at 1 year of age.

Of the original 14,541 initial pregnancies, 338 were from a woman who had already enrolled with a previous pregnancy, meaning 14,203 unique mothers were initially enrolled in the study. As a result of the additional phases of recruitment, a further 630 women who did not enroll originally have provided data since their child was 7 years of age. This provides a total of 14,833 unique women (G0 mothers) enrolled in ALSPAC as of September 2021.

G0 partners were invited to complete questionnaires by the mothers at the start of the study and they were not formally enrolled at that time. 12,113 G0 partners have been in contact with the study by providing data and/or formally enrolling when this started in 2010. 3,807 G0 partners are currently enrolled.

Informed consent for the use of data collected via questionnaires and clinics was obtained from participants following the recommendations of the ALSPAC Ethics and Law Committee at the time.

# Supplementary text 2 | Mediterranean Diet score calculation

The Mediterranean diet score was composed of the 7 food groups or components. Each component consisted of a varying number of relevant items (reported in grams per day) which were summed together to obtain mean daily consumption.

Beneficial components:

1. *Cereals*: including 3 items in ALSPAC (“Brown and granary bread”, “Wholemeal bread”, and “High fiber breakfast cereals”) and 3 items in GenR (“Brown/wholegrain bread”, “[Other] wholegrain products”, and “Porridge”).
2. *Fish*: including 2 items in ALSPAC (“[Non-fried] white fish, shellfish, fish dishes”, and “Oily fish”) and 5 items in GenR (“Fatty fish (>10% fat)”, “Lean fish (<2% fat)”, “Moderately fat fish (2-10%)”, “Shellfish”, and “Canned fatty fish”).
3. *Vegetables*: including 6 items in ALSPAC (“Raw carrots”, “Cooked carrots”, “Green leafy vegetables”, “Other salad and raw vegetables”, “Other cooked vegetables”, and “Vegetable dishes”) and 2 items in GenR (“Raw vegetables”, and “Cooked vegetables”).
4. *Legumes*: including 4 items in ALSPAC (“Baked beans”, “Peas”, “Green and runner beans”, and “[Other] legumes”) and 1 item in GenR (“Canned pulses”).
5. *Fruit*: including 8 items in ALSPAC (“Apples and pears”, “Bananas”, “Citrus fruit”, “Raw tomatoes”, “Cooked and canned tomatoes”, “Fruit canned in juice”, “Other fruit”, and “Nuts”) and 3 items in GenR (“Fresh fruit”, “Dried fruit (raisins)” and “Nuts, unsalted”).

Detrimental components:

1. *Meat*: including 12 items in ALSPAC (“Coated chicken and turkey”, “Chicken, turkey and dishes”, “Lamb and lamb dishes”, “Pork and pork dishes”, “Beef and beef dishes”, “Liver and liver dishes”, “Burgers and kebabs”, “Sausages”, “Offal (excluding liver)”, “Other meat and meat products”, “Ham and bacon”, and “Butter”) and 8 items in GenR (“Processed red meat”, “Processed white meat”, “Unprocessed red meat, high-fat (>5% saturated fatty acids (SFA))”, “Unprocessed red meat, low-fat (<=5% SFA)”, “Unprocessed white meat, low-fat (<=5% SFA)”, “Sausage rolls (e.g., worstebroodje, sausijzenbroodje)”, “Fast food meat (e.g., kroket, frikandel)”, and “Hard fats, butter”).
2. *Dairy*: including 6 items in ALSPAC (“Cheese”, “Whole milk”, “Semi-skimmed milk”, “Skimmed milk”, “Goat and sheep milk”, “Yoghurt and fromage frais”) and 8 items in GenR (“Full-fat cheese (> 30+)”, “Low-fat cheese (<=30+)”, “Full-fat milk”, “Full-fat yoghurt or quark”, “Skimmed or semi skimmed milk, buttermilk, with no added sugar”, “Skimmed or semi-skimmed yoghurt or quark, with no added sugar”, “Milk-based beverages with added sugar”, and “Yoghurt or quark with added sugar”).

# Supplementary text 3 | Imputation rationale and quality

**Detailed strategy description**

Missing values were imputed by fully conditional multiple imputation^1^, using 60 iterations and 30 imputed datasets, as implemented by the mice package (version 3.13.0)^2^. The method of the imputation model was set to *predictive mean matching*, which is the recommended option in order to take into account non-normality (as well as sparse categorical data)^1,3^, and it is relatively robust against model misspecification^1,4^.

The auxiliary variables selected for the imputation model included: maternal age and smoking during pregnancy, gestational age and weight of the child at birth, parity, ethnicity and sex of the child, BMI of the mother during pregnancy and during childhood (measured when the child was 5), and maternal depressive symptoms during pregnancy and during childhood (measured when the child was 3).

Auxiliary variables were selected because they are believed to be either related to missingness or to the domain scores and primary outcomes themselves. When auxiliary information was available both prenatally and postnatally, the opposite period was used in the stress variables’ imputation, in order to minimize bias. E.g., for the imputation of prenatal items, we used BMI of the mother when the child was 5, and for imputation of postnatal items we used BMI of the mother during pregnancy. This approach was meant to both reduce computational load and avoid multicollinearity issues.

Exposure imputation

Prenatal and postnatal stress scores were imputed in three steps: first the individual items were imputed according to the model specified below. Then mean domain scores were “passively” derived by averaging these complete indicators within their respective domain. At this stage a 25% cut-off was applied so that if less than 25% of the domain items was missing, only observed values were used, otherwise the imputed values were included in the calculation. Finally, the total prenatal and postnatal scores were calculated by summing the obtained domain scores within their respective periods.

The imputation of single ELS items was based on the following information:

1. all other ELS items belonging to the same domain,
2. other domain scores (excluding the domain of the imputed item),
3. all auxiliary variables except for those that were part of the domain.

Covariates’ imputation

Missing model covariates (age and ethnicity of the child, maternal BMI before pregnancy, maternal smoking and drinking behavior during pregnancy) were imputed given the outcome variables and the domain scores.

Outcome imputation

The primary outcomes (internalizing and adiposity at age 13) were imputed based on the total prenatal and postnatal stress scores, previous measurements of the same or correlated outcomes (assessed when the children were 10 years old), and the auxiliary variables.

Finally, the secondary “risk group” outcome (meat as a proxy of comorbidity) was imputed passively using the complete primary outcomes, as described in the main manuscript.

Moderators’ imputation

Missing values in each lifestyle factor moderator variable was imputed using the total ELS score, model covariates, each outcome of interest, the other moderators and all available repeated measurements of each moderator. These were different in the two cohorts.

In the ALSPAC sample:

- Physical activity frequency (at 10.7 years) was imputed using physical activity frequency at 8.2, 9.7, 11.7, 13.2, 14.7, 15.4, 16.1, and 17 years of age.
- Sleep duration (at 11 years) was imputed using sleep duration at 4.8, 5.8, 6.8 and 9.7 years.
- Mediterranean diet score (at 10.7 years) was imputed using Mediterranean diet score at 7.5 years.

In the Generation R sample:

- Physical activity frequency (at 9.8 years) was imputed using information about frequency of participation in sports at 6 and 9.7 years, frequency of walking or cycling to school (at 6 and 9.7 years), and hours of outdoor play (at 4 and 9.7 years).
- Sleep duration (at 11.8 / 14 years) was imputed using maternal reports of sleep problems (i.e., CBCL sleep problems subscale) at 1.5, 3, and 6 years.
- No repeated measurements were available for Mediterranean diet score.

Exclusion criteria

We adopted a partial “imputation then deletion” approach for the exposure variables, whereas the imputed outcomes values were retained in the main analysis.

Participants were excluded from analysis when the frequency of missing ELS variables in the prenatal or the postnatal period exceeded 50%. All twins were further excluded. Finally, only one sibling was selected based on data completeness, or, if that was equal between the siblings, randomly.

**Theoretical rationale**

There are two important potential consequences of missing data. The first is the decrease in *precision* (wider confidence intervals) and *power* caused by the reduction in data. The second, and more serious, is the potential for *bias* in the estimation of association parameters^5-7^.

Primer: missing data mechanisms

The complexity of the missing data problem, i.e., obtaining accurate inferential estimates in the presence of missing data, depends on the nature of the mechanism by which data are missing ^5^. Little and Rubin ^5,8^ provided a popular framework to describe categories of missing data mechanisms given the relationship with observed and unobserved values.

The less problematic scenario occurs when the probability of an observable data point being missing does not depend on any observed or unobserved parameters: Missing Completely at Random (MCAR). More commonly, the missingness probability depends on observed variables, and hence it can be accounted for by the information contained in the dataset: Missing at Random (MAR). The most challenging missingness mechanism occurs when the missingness probability depends on unobserved values: Missing Not at Random (MNAR).

Unfortunately, it is not possible to distinguish between MAR and MNAR mechanisms without additional external data or prior knowledge. However, the MAR assumption is usually reasonable in the context of longitudinal observational studies ^7,9^.

It is important to realize that the term *missing at random* does not mean that the missing data are a simple random subsample of all the data points. That scenario is MCAR. Under MAR, missing data may be more frequent in some subgroups than in others, but information defining the subgroups is observed ^9^.

Although missing data has the potential to cause serious bias, it is still possible to perform a valid and sensible analysis. Among the various available approaches to handling missing data^6,8,10^, multiple imputation (MI) has been widely adopted and accepted by methodologists as an appropriate framework for dealing with MAR (and MCAR) mechanisms^1,5-7,9-11^.

Primer: Multiple imputation

The multiple imputation approach adopted in this study can be described in three steps:

(1) Create multiple (*M*=30) copies of the dataset, with the missing values replaced by imputed values. To determine these values, the regression models described above are used to find cases with observed data that have predicted values closely resembling the predicted values of the respondents with missing values.

(2) Analyse each dataset separately with the chosen method (i.e., linear and multinomial logistic regression model, and causal mediation analysis).

(3) Pool the estimates and their standard errors across the *M* analyses using Rubin’s rules^5^, that allow to take into account the within-imputation and between-estimation variation components in the calculations. In this way, the uncertainty associated with imputation is accounted for.

The implicit and un-testable assumption is that the relationship between observed and missing values is the same for those who complete and those who do not: i.e., the MAR assumption.

A partial reason for MI’s success is its flexibility^6^. MI is the only approach that can be used with any analytic model^1^, which was particularly useful considered the range of variables and analytical approaches involved in this study.

Moreover, one of the most appealing features of the MI framework is the ability to incorporate additional “auxiliary” variables into the imputation model to improve the prediction of missing values. Incorporation of auxiliary data can make assumptions about the ignorability of the missing data more likely by reducing (or eliminating) bias^9,12^. The guiding principle in the construction of the above-specified models was indeed to leverage a wide range of information sources that could be predictive of the missing values themselves or influencing the process causing the missing data, even when these variables were not of interest in the substantive analysis.

For the imputation of exposure variables, the approach we have chosen involves two adaptations of the classical multiple imputation model: we defined a domain-specific set of predictors for individual item and we included a stepwise passive imputation for domain and total scores. This approach was recommended by van Buuren^1^ as it has been found to reduce standard error substantially compared to complete-case analysis ^13^, and to outperform other existing techniques for handling large multi-item scales ^14^.

The outcome was included in the imputation model of missing covariate values, as recommended ^9,15^, however it was not included in the imputation of the stress predictors, although it might have improved the imputation performance ^16^. This was done with the aim of creating a more generally valid stress score that could be used with a variety of outcomes in future studies, however this should be noted as a limitation.

As per the handling of missing outcome values, this was a particularly sensitive issue in this study, given the rather extended follow-up time that separated the baseline cohort inclusion from the outcome measurements (i.e., ~13 years). Unfortunately, discussion of these issues is lacking in the current literature and it is often unclear how missing data are being handled in practice in this context ^6,17-19^. However, few simulation studies have shown that using information on exposure and correlated (e.g., longitudinally measured) outcomes in the imputation model reduced bias compared to alternatives^11,20-22^ such as deleting cases with missing data or imputing and then deleting^21^.

More broadly, although simpler solutions for handling missing outcomes are still routinely used^6,9,17-19^, these approaches have been amply shown to be inadequate and even misleading^7,9-11,18,19^, as they do not preserve important characteristics of the whole data set, such as key relationships among the variables and means^9,19^. For an overview of simulation studies that confirm that multiple imputation is a better alternative than listwise deletion and single imputation consult Van Buuren (2018)^1^.

For instance, list-wise deletion (e.g., selecting on outcome availability prior to imputation) *requires MCAR data* in order to not introduce bias in the results^6,9^, it makes strong assumptions about the covariance structure of the data (that it is compound symmetric), and it has been discouraged by statisticians ^1,9^. In our study, we have reason to believe that dropout might depend at least partly on measured (or unmeasured) variables. For example, families that are exposed to lower levels of stress may be more likely to return follow-up questionnaires and bring their children to the visits. If that was indeed the case, the MCAR assumption would be violated and important concerns for selection bias would arise.

Deleting imputed outcomes prior to analysis can also lead to bias, especially when the imputation model contains variables that are associated with missingness in the outcome^21^, as just described.

Hence, although it is important to point out that our approach relies on MAR assumptions, and we cannot guarantee unbiased results under the situation where missingness depends on unobserved information (MNAR), these assumptions are not nearly as strong (or in some cases, as unrealistic) as those required for a complete-case analysis. Moreover, it has been argued that MI can offer some protection against MNAR mechanisms ^9,23^, unlike MCAR methods ^24^, although this has not been quantified within a simulation framework. Nevertheless, MI can accommodate MNAR scenarios flexibly and is thus is well-suited to sensitivity analyses^25^.

**Missing patterns and imputation quality results**

Table 4.1 provides some key variables’ descriptives before (i.e., in the original sample) and after imputation (i.e., pooled across the 30 imputed datasets) together with the number and percentage of missing values in both cohorts.

Note that the pooled means (i.e., after imputation) of the exposure components and of the primary outcomes are slightly higher compared to the original metrics. We believe that this upward shift is to be expected under the assumption that the group lost to follow-up may be more likely to experience higher levels of stress, and higher physical and/or psychological problems. None of these differences resulted statistically significant.

The fraction of missing values per variable ranges from 0 to a maximum of 51% (i.e., the risk group variable in the ALSPAC cohort). Although the level of missingness naturally affects MI performance, we decided to leverage all available data even when missingness was extensive, as recommended by several statisticians ^11,15,26^.

In support of this approach, two simulation studies also suggested that, under the condition of a large enough sample size (<1000) and given that the imputation model is appropriate, such levels of missingness still allow for a reasonably low expected bias if any^11,22^.

Convergence

Visual inspection of the convergence graphs showed no signs of unhealthy convergence. In some cases, the trace lines showed strong initial trends and slow mixing, but regardless of the proportion of missing data, results were stable after 20-30 of the 60 iterations. The convergence plots are not presented here, but are available upon request to SD.

Imputed vs. observed values

Next, we inspected and compared the density of the incomplete and imputed data. These graphs are publickly available for the key variables of interest and in the two cohorts (see [GitHub repository](https://github.com/SereDef/lifestyle-moderators-project)). The blue line marks the observed and red lines indicate imputed values.

Finally, to detect possible issues with the passive computation of the stress domain scores, we inspected the calculated domain values against imputed values These graphs are also publickly available for the key variables of interest and in the two cohorts (see [GitHub repository](https://github.com/SereDef/lifestyle-moderators-project)).

**Sensitivity analysis**

A thorough and sensible sensitivity analysis is an important step in producing and reporting robust estimates ^6^. Hence, we also included a sensitivity analysis to assess the extent to which analytic approaches are robust to missing data assumptions. None of the main conclusions were impacted.

#
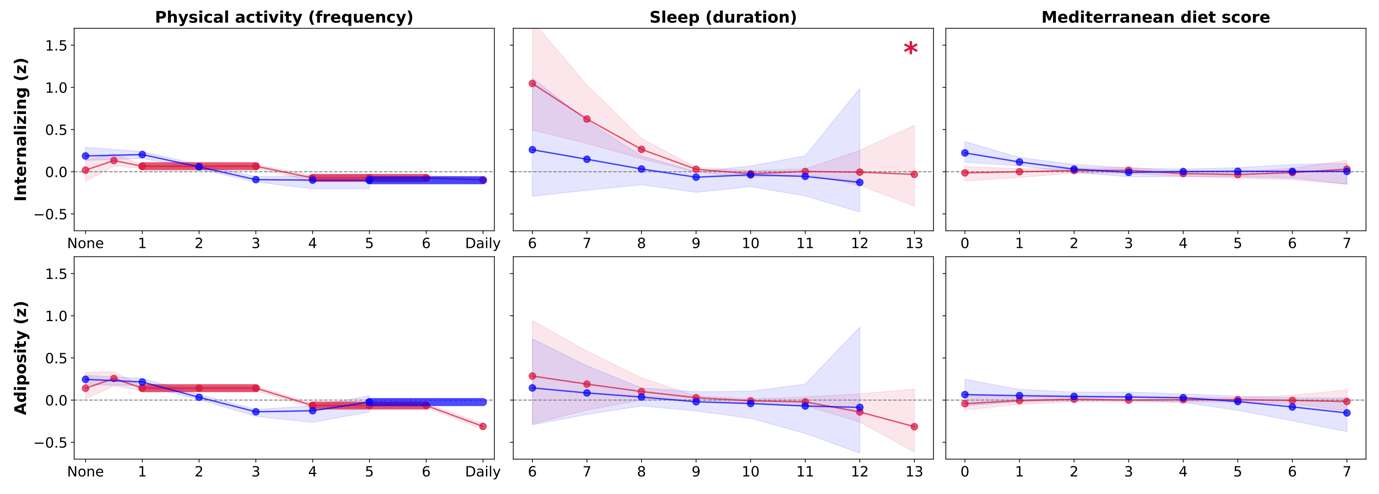
Figure S1 | Non-linear relations

Each moderator was modelled using natural splines with 3 knots and pooled across 30 imputed datasets for GenR (blue) and ALSPAC (red). The asterisk indicates a significantly better fit of the non-linear model compared to the linear one in at least half of the imputed sets.

# Supplementary references

1. Van Buuren S. *Flexible imputation of missing data*. CRC press; 2018.

2. van Buuren S, Groothuis-Oudshoorn K. mice: Multivariate Imputation by Chained Equations in R. *Journal of Statistical Software*. 12/12 2011;45(3):1 - 67. doi:10.18637/jss.v045.i03

3. Marshall A, Altman DG, Royston P, Holder RL. Comparison of techniques for handling missing covariate data within prognostic modelling studies: a simulation study. *BMC Medical Research Methodology*. 2010/01/19 2010;10(1):7. doi:10.1186/1471-2288-10-7

4. Van Buuren S, Brand JP, Groothuis-Oudshoorn CG, Rubin DB. Fully conditional specification in multivariate imputation. *Journal of statistical computation and simulation*. 2006;76(12):1049-1064.

5. Rubin DB. Multiple imputation for survey nonresponse. New York: Wiley; 1987.

6. Bell ML, Fairclough DL. Practical and statistical issues in missing data for longitudinal patient-reported outcomes. *Stat Methods Med Res*. Oct 2014;23(5):440-59. doi:10.1177/0962280213476378

7. Sterne JA, White IR, Carlin JB, et al. Multiple imputation for missing data in epidemiological and clinical research: potential and pitfalls. *BMJ (Clinical research ed)*. 2009;338

8. Little RJ, Rubin DB. *Statistical analysis with missing data*. vol 793. John Wiley & Sons; 2019.

9. van Ginkel JR, Linting M, Rippe RCA, van der Voort A. Rebutting Existing Misconceptions About Multiple Imputation as a Method for Handling Missing Data. *Journal of Personality Assessment*. 2020/05/03 2020;102(3):297-308. doi:10.1080/00223891.2018.1530680

10. Graham JW, Cumsille PE, Shevock AE. Methods for handling missing data. *Handbook of psychology: Research methods in psychology, Vol 2, 2nd ed*. John Wiley & Sons, Inc.; 2013:109-141.

11. Kontopantelis E, White IR, Sperrin M, Buchan I. Outcome-sensitive multiple imputation: a simulation study. *BMC Medical Research Methodology*. 2017/01/09 2017;17(1):2. doi:10.1186/s12874-016-0281-5

12. Wang C, Hall CB. Correction of bias from non-random missing longitudinal data using auxiliary information. *Statistics in Medicine*. 2010;29(6):671-679. doi:<https://doi.org/10.1002/sim.3821>

13. Plumpton CO, Morris T, Hughes DA, White IR. Multiple imputation of multiple multi-item scales when a full imputation model is infeasible. *BMC Res Notes*. 2016;9:45-45. doi:10.1186/s13104-016-1853-5

14. Eekhout I, de Vet HC, de Boer MR, Twisk JW, Heymans MW. Passive imputation and parcel summaries are both valid to handle missing items in studies with many multi-item scales. *Statistical methods in medical research*. 2018;27(4):1128-1140.

15. Janssen KJ, Donders ART, Harrell Jr FE, et al. Missing covariate data in medical research: to impute is better than to ignore. *Journal of clinical epidemiology*. 2010;63(7):721-727.

16. Moons KG, Donders RA, Stijnen T, Harrell Jr FE. Using the outcome for imputation of missing predictor values was preferred. *Journal of clinical epidemiology*. 2006;59(10):1092-1101.

17. Wood AM, White IR, Thompson SG. Are missing outcome data adequately handled? A review of published randomized controlled trials in major medical journals. *Clin Trials*. 2004;1(4):368-76. doi:10.1191/1740774504cn032oa

18. Peugh JL, Enders CK. Missing Data in Educational Research: A Review of Reporting Practices and Suggestions for Improvement. *Review of Educational Research*. 2004;74(4):525-556. doi:10.3102/00346543074004525

19. Jelicić H, Phelps E, Lerner RM. Use of missing data methods in longitudinal studies: the persistence of bad practices in developmental psychology. *Dev Psychol*. Jul 2009;45(4):1195-9. doi:10.1037/a0015665

20. Floden L, Bell ML. Imputation strategies when a continuous outcome is to be dichotomized for responder analysis: a simulation study. *BMC Medical Research Methodology*. 2019/07/23 2019;19(1):161. doi:10.1186/s12874-019-0793-x

21. Sullivan TR, Salter AB, Ryan P, Lee KJ. Bias and Precision of the “Multiple Imputation, Then Deletion” Method for Dealing With Missing Outcome Data. *American Journal of Epidemiology*. 2015;182(6):528-534. doi:10.1093/aje/kwv100

22. Demirtas H, Freels SA, Yucel RM. Plausibility of multivariate normality assumption when multiply imputing non-Gaussian continuous outcomes: a simulation assessment. *Journal of Statistical Computation and Simulation*. 2008/02/01 2008;78(1):69-84. doi:10.1080/10629360600903866

23. Schafer JL, Graham JW. Missing data: our view of the state of the art. *Psychological methods*. 2002;7(2):147.

24. Molenberghs G, Thijs H, Jansen I, et al. Analyzing incomplete longitudinal clinical trial data. *Biostatistics*. Jul 2004;5(3):445-64. doi:10.1093/biostatistics/5.3.445

25. Groenwold RH, Donders ART, Roes KC, Harrell Jr FE, Moons KG. Dealing with missing outcome data in randomized trials and observational studies. *American journal of epidemiology*. 2012;175(3):210-217.

26. Madley-Dowd P, Hughes R, Tilling K, Heron J. The proportion of missing data should not be used to guide decisions on multiple imputation. *Journal of Clinical Epidemiology*. 2019/06/01/ 2019;110:63-73. doi:<https://doi.org/10.1016/j.jclinepi.2019.02.016>
